# On Generalized Carleson Operators of Periodic Wavelet Packet Expansions

**DOI:** 10.1155/2013/379861

**Published:** 2013-08-20

**Authors:** Shyam Lal, Manoj Kumar

**Affiliations:** Department of Mathematics, Faculty of Science, Banaras Hindu University, Varanasi 221005, India

## Abstract

Three new theorems based on the generalized Carleson operators for the periodic Walsh-type wavelet packets have been established. An application of these theorems as convergence a.e. for the periodic Walsh-type wavelet packet expansion of block function with the help of summation by arithmetic means has been studied.

## 1. Introduction

Wavelet packet expansions have wide applications in engineering and technology. The Walsh-type wavelet packet expansions play an important role in signal processing, numerical analysis, and quantum mechanics. A family of nonstationary wavelet packets considered the smooth generalization of the Walsh functions having some of the same nice convergence properties for expansion of *L* ^*p*^-function, 1 < *p* < *∞*, as the Walsh-Fourier series. Walsh-type wavelet packet expansion has been studied by the researchers Billard [1], Nielsen [2], Sjölin [3] and others. In 1966, at first, Carleson operator has been introduced by Lennart Carleson (Carleson [4]). Several important properties of this operator has been studied by researcher Nielsen [2]. In this paper, the pointwise convergence almost everywhere by arithmetic means or (*C*, 1) summability method of the partial sum operator for Walsh-type wavelet packet expansion of functions from the block space, *𝔹*_*q*_,  1 < *q* ≤ *∞*,  *p* ^−1^ + *q* ^−1^ = 1 has been studied. Generalized Carleson operators are introduced and some new properties of generalized Carleson operators are investigated. Specific convergence properties of Walsh-type wavelet packet expansions of block functions using (*C*, 1) method and generalized Carleson operator have been obtained.

## 2. Definitions and Preliminaries

*Walsh-Type Wavelet Packets. *To every multiresolution analysis {*V*_*j*_}_*j*∈*ℤ*_ for *L* ^2^(ℝ), an associated scaling function *φ* and a wavelet *ψ* are given with the properties that

(1) $$\begin{array}{c} V_{j}=\bar{\mathrm{span}}\left\{2^{j/2}\varphi \left(2^{j}\cdot -k\right):k\in \mathbb{Z}\right\},\;j\in \mathbb{Z},\\ \left\{\psi_{j,k}\equiv 2^{j/2}\psi \left(2^{j}\cdot -k\right):j,k\in \mathbb{Z}\right\} \end{array}$$

is an orthonormal basis for *L* ^2^(ℝ).

We write

(2) $$\begin{array}{c} W_{j}=\bar{\mathrm{span}}\left\{2^{j/2}\psi \left(2^{j}\cdot -k\right):k\in \mathbb{Z}\right\},\;j\in \mathbb{Z}. \end{array}$$

Let *ℕ* be the set of natural numbers. Let (*F*_0_^(*p*)^, *F*_1_^(*p*)^), *p* ∈ *ℕ*, be a family of bounded operators on *l* ^2^(*ℤ*) of the form

(3) $$\begin{array}{c} {\left(F_{\epsilon }^{\left(p\right)}a\right)}_{k}=\sum_{n\in \mathbb{Z}}a_{n}h_{\epsilon }^{\left(p\right)}\left(n-2k\right),\;\epsilon =0,1 \end{array}$$

with *h*_1_^(*p*)^(*n*) = (−1)^*n*^ *h*_0_^(*p*)^(1 − *n*) a real-valued sequence in *l* ^1^(*ℤ*) such that

(4) $$\begin{array}{c} F_{0}^{(p)*}F_{0}^{\left(p\right)}+F_{1}^{(p)*}F_{1}^{\left(p\right)}=1,\\ F_{0}^{\left(p\right)}F_{1}^{(p)*}=0. \end{array}$$

Define the family of functions {*w*_*n*_}_*n*=0_^*∞*^ recursively by letting *w*_0_ = *φ*,   *w*_1_ = *ψ* and then for *n* ∈ *ℕ*,

(5) $$\begin{array}{c} w_{2n}\left(x\right)=\sqrt{2}\sum_{l\in \mathbb{Z}}h_{0}^{\left(p\right)}\left(l\right)w_{n}\left(2x-l\right),\\ w_{2n+1}\left(x\right)=\sqrt{2}\sum_{l\in \mathbb{Z}}h_{1}^{\left(p\right)}\left(l\right)w_{n}\left(2x-l\right), \end{array}$$

where 2^*p*^ ≤ *n* < 2^*p*+1^.

The family {*w*_*n*_}_*n*=0_^*∞*^ is basic non stationary wavelet packets. {*w*_*n*_(·−*k*) : *n* ≥ 0, *k* ∈ *ℤ*} is an orthonormal basis for *L* ^2^(ℝ).

Moreover,

(6) $$\begin{array}{c} \left\{w_{n}\left(\cdot -k\right):2^{j}\leq n<2^{j+1},k\in \mathbb{Z}\right\} \end{array}$$

is an orthonormal basis for $W_{j}=\bar{\mathrm{span}}\left\{2^{j/2}\psi (2^{j}\cdot -k):k\in \mathbb{Z}\right\}$.

Each pair (*F*_0_^(*p*)^, *F*_1_^(*p*)^) can be chosen as a pair of quadrature mirror filters associated with a multiresolution analysis, but this is not necessary.

The trigonometric polynomials given by

(7) $$\begin{array}{c} m_{0}^{\left(p\right)}\left(\xi \right)=\frac{1}{\sqrt{2}}\sum_{k\in \mathbb{Z}}h_{0}^{\left(p\right)}\left(k\right)e^{-ik\xi },\\ \;\;m_{1}^{\left(p\right)}\left(\xi \right)=\frac{1}{\sqrt{2}}\sum_{k\in \mathbb{Z}}h_{1}^{\left(p\right)}\left(k\right)e^{-ik\xi } \end{array}$$

are called the symbols of the filters.

The Fourier transforms of (5) are given by

(8) $$\begin{array}{c} {\hat{w}}_{2n}\left(\xi \right)=m_{0}^{\left(p\right)}\left(\frac{\xi }{2}\right){\hat{w}}_{n}\left(\frac{\xi }{2}\right),\\ {\hat{w}}_{2n+1}\left(\xi \right)=m_{1}^{\left(p\right)}\left(\frac{\xi }{2}\right){\hat{w}}_{n}\left(\frac{\xi }{2}\right). \end{array}$$

The Haar low-pass quadrature mirror filter {*h*_0_(*k*)}_*k*_ is given by $h_{0}(0)=h_{0}(1)=1/\sqrt{2},\;\;h_{0}(k)=0$ otherwise, and the associated high-pass filter {*h*_1_(*k*)}_*k*_ is given by

(9) $$\begin{array}{c} h_{1}\left(k\right)={\left(-1\right)}^{k}h_{0}\left(1-k\right). \end{array}$$

**Definition 1**

Let {*w*_*n*_}_*n*≥0,*k*∈*ℤ*_ be a family of non-stationary wavelet packets constructed by using a family {*h*_0_^(*p*)^(*n*)}_*p*=1_^*∞*^ of finite filters for which there is a constant, *K* ∈ *ℤ* such that *h*_0_^(*p*)^(*n*) is the Haar filter for every *p* ≥ *K*. If *w*_1_ ∈ *C* ^1^(ℝ) is compactly supported then {*w*_*n*_}_*n*≥0_ is called a family of Walsh-type wavelet packets.

**Definition 2**

Let {*w*_*n*_}_*n*=0_^*∞*^ be a family of Walsh-type basic wavelet packets. For *n* ∈ *ℕ*_0_, define the corresponding periodic Walsh-type wavelet packets ${\tilde{w}}_{n}$ by

(10) $$\begin{array}{c} {\tilde{w}}_{n}\left(x\right)=\sum_{k\in \mathbb{Z}}w_{n}\left(x-k\right). \end{array}$$

From Fubini's theorem, it follows that ${\left\{{\tilde{w}}_{n}\right\}}_{n=0}^{\infty }$ is an orthonormal basis for *L* ^2^[0,1).

*Block Spaces.* A dyadic *q*-block is a function *β* ∈ *L* ^*q*^[0,1) which is supported on some dyadic interval *I* such that ||*β*||_*q*_ ≤ |*I*|^1/*q*−1^, where ||*β*||_*q*_ = [∫_0_^1^|*β*(*t*)|^*q*^ *dt*]^1/*q*^, 1 < *q* < *∞*. Let *𝔹*_*q*_ denote the space of measurable functions *f* on [0,1) which has an expansion

(11) $$\begin{array}{c} f=\sum_{k=1}^{\infty }c_{k}\beta_{k}, \end{array}$$

where each *β*_*k*_ is a *q*-block and the coefficients *c*_*k*_, *k* ∈ *ℤ* satisfy

(12) $$\begin{array}{c} ||\left|\left\{c_{k}\right\}\right|||=\sum_{k:c_{k}\neq 0}\left|c_{k}\right|\left[1+\log\frac{\sum_{j=1}^{\infty }\left|c_{j}\right|}{\left|c_{k}\right|}\right]<\infty . \end{array}$$

The quasi norm of *f* ∈ *𝔹*_*q*_ is given as the infimum of |||·||| over all possible decompositions of *f* into blocks

(13) $$\begin{array}{c} {||f||}_{{𝔹}_{q}}=\underset{f=\sum c_{k}\beta_{k}}{\inf}||\left|\left\{c_{k}\right\}\right|||. \end{array}$$

Let *f* ∈ *𝔹*_*q*_; then

(14) $$\begin{array}{c} {||f||}_{1}\leq \sum_{k=1}^{\infty }\left|c_{k}\right|{||\beta_{k}||}_{1}\leq \sum_{k=1}^{\infty }\left|c_{k}\right|<\infty , \end{array}$$

using (12) and the fact that for each *k*,  ||*β*_*k*_||_*q*_ ≤ |*I*|^1/*q*−1^ which implies that ||*β*_*k*_||_1_ ≤ 1; that is, *𝔹*_*q*_ ⊂ *L* ^1^[0,1). Moreover, for

(15) $$\begin{array}{c} f\in L^{q}\left[0,1\right),\;1<q<\infty ,\;\beta ={||f||}_{q}^{-1}f \end{array}$$

is a *q*-block supported on *I* = [0,1) so *L* ^*q*^[0,1) ⊂ *𝔹*_*q*_.

The classical example to show that for each *q* > 1 there exists *f* ∈ *𝔹*_*q*_ which belongs to none of the *L* ^*p*^[0,1)-space is the following.

Let

(16) $$\begin{array}{c} \beta_{k}\left(x\right)=\{\begin{array}{cc} 2^{k}, & \frac{1}{2^{k}}<x\leq \frac{3}{2^{\left(k+1\right)}},\\ 0, & \text{otherwise}. \end{array} \end{array}$$

Then *f* = ∑_*k*=1_^*∞*^ *k* ^−2^ *β*_*k*_ ∈ *𝔹*_*q*_, but ||*f*||_*p*_^*p*^ = ∑_*k*=1_^*∞*^(1/2)*k* ^−2*p*^2^*k*(*p*−1)^ = *∞* for every *p* > 1.

*Summation of Series by Arithmetic Means.* If a series *u*_0_ + *u*_1_ + *u*_2_ + ⋯ is not convergent, that is, if *s*_*n*_ = *u*_0_ + *u*_1_ + *u*_2_ + ⋯+*u*_*n*_ does not tend to a limit, it is some time possible to associate with the series a “sum” in a less direct way. The simplest such method is “summation by arithmetic means”. Let

(17) $$\begin{array}{c} \sigma_{n}=\frac{s_{0}+s_{1}+s_{2}+\cdots +s_{n}}{n+1} \end{array}$$

be the arithmetic mean of the partial sums of the given series.

If *s*_*n*_ → *s*, then also *σ*_*n*_ → *s*; for if *s*_*n*_ = *s* + *δ*_*n*_, then

(18) $$\begin{array}{c} \sigma_{n}=s+\frac{\delta_{0}+\delta_{1}+\delta_{2}+\delta_{2}+\cdots +\delta_{n}}{n+1}, \end{array}$$

and the last term tends to zero if *δ*_*n*_ → 0. Consider

(19) σn=s0+s1+s2+⋯+snn+1=(u0+(u0+u1)+⋯+(u0+u1+⋯+uk) +⋯+(u0+u1+⋯+un))×(n+1)−1=∑k=0n(1−kn+1)uk.

If *σ*_*n*_ → *s* as *n* → *∞*, ∑_*n*=0_^*∞*^ *u*_*n*_ is said to be summable to *s* by Cesàro's means of order 1. We write

(20) $$\begin{array}{c} \sum_{n=0}^{\infty }u_{n}=s\left(C,1\right). \end{array}$$

But *σ*_*n*_ may tend to a limit even though *s*_*n*_ does not, for example, the series

(21) $$\begin{array}{c} 1-1+1-1+\cdots . \end{array}$$

Here the partial sums *s*_*n*_ are alternately 1 and 0, and it is easily seen that *σ*_*n*_ → 1/2.

### 2.1. Generalized Carleson Operators

Let $\left\{{\tilde{w}}_{n}\right\}$ be a periodic Walsh-type wavelet packet basis. For any function *f* ∈ *L* ^1^[0,1), define

(22) $$\begin{array}{c} \left(S_{N}f\right)\left(x\right)=\sum_{n=0}^{N}\langle f,{\tilde{w}}_{n}\rangle {\tilde{w}}_{n}\left(x\right). \end{array}$$

The Carleson operator *𝔾* is defined by

(23) 𝔾f(x)=sup⁡N≥0|∑n=0N〈f,w~n〉w~n(x)|=sup⁡N≥0|(SNf)(x)|.

The generalized Carleson operator *𝔾*_*c*_ is defined by

(24) 𝔾cf(x)=sup⁡N≥0|(S0f)(x)+(S1f)(x)+⋯+(SNf)(x)N+1|=sup⁡N≥0|1N+1∑ν=0N‍ ∑n=0ν〈f,w~n〉w~n(x)|=sup⁡N≥0|∑n=0N(1−nN+1)〈f,w~n〉w~n(x)|.

The weak Carleson operator *G* is defined by

(25) Gf(x)=limsup⁡N≥0|∑n=0N〈f,w~n〉w~n(x)|.=limsup⁡N≥0|(SNf)(x)|.

The generalized weak Carleson operator *G*_*c*_ is define by

(26) Gcf(x)=limsup⁡N≥0|(S0f)(x)+(S1f)(x)+⋯+(SNf)(x)N+1|=limsup⁡N≥0|1N+1∑ν=0N‍ ∑n=0ν〈f,w~n〉w~n(x)|=limsup⁡N≥0|∑n=0N(1−nN+1)〈f,w~n〉w~n(x)|.

The dyadic Carleson operator *𝔾* ^*d*^ is defined by

(27) 𝔾df(x)=sup⁡N≥0|∑n=02N−1〈f,w~n〉w~n(x)|=sup⁡N≥0|(S2Nf)(x)|.

The generalized dyadic Carleson operator *𝔾*_*c*_^*d*^ is define by

(28) 𝔾cdf(x)=sup⁡N≥0|(S0f)(x)+(S1f)(x)+⋯+(S2N−1f)(x)2N|=sup⁡N≥0|12N∑ν=02N−1‍ ∑n=0ν〈f,w~n〉w~n(x)|=sup⁡N≥0|∑n=02N−1(1−n2N)〈f,w~n〉w~n(x)|.

It is easy to prove that *𝔾*_*c*_, *G*_*c*_ and *𝔾*_*c*_^*d*^ are sublinear operators.

*Walsh Functions and Their Properties.* The Walsh system {*W*_*n*_}_*n*=0_^*∞*^ is defined recursively on [0,1) by letting

(29) $$\begin{array}{c} W_{0}\left(x\right)=\{\begin{array}{cc} 1, & 0\leq x<1;\\ 0, & \text{otherwise}, \end{array}\\ W_{2n}\left(x\right)=W_{n}\left(2x\right)+W_{n}\left(2x-1\right),\\ W_{2n+1}\left(x\right)=W_{n}\left(2x\right)-W_{n}\left(2x-1\right). \end{array}$$

Observe that the Walsh system is the family of wavelet packets obtained by considering *φ* = *W*_0_,

(30) $$\begin{array}{c} \psi \left(x\right)=\{\begin{array}{cc} 1, & 0\leq x<\frac{1}{2};\\ -1, & \frac{1}{2}\leq x<1;\\ 0, & \text{otherwise} \end{array} \end{array}$$

and using the Haar filters in the definition of the nonstationary wavelet packets.

The Walsh system is closed under pointwise multiplication. Define the binary operator ⊕:*ℕ*_0_ × *ℕ*_0_ → *ℕ*_0_ by

(31) $$\begin{array}{c} m⊕n=\sum_{i=0}^{\infty }\left|m_{i}-n_{i}\right|2^{i}, \end{array}$$

where *m* = ∑_*i*=0_^*∞*^ *m*_*i*_2^*i*^ and *n* = ∑_*i*=0_^*∞*^ *n*_*i*_2^*i*^. Then

(32) $$\begin{array}{c} W_{m}\left(x\right)W_{n}\left(x\right)=W_{m⊕n}\left(x\right), \end{array}$$

(see Schipp et al. [5]). 

 We can carry over the operator ⊕ to the interval [0,1] by identifying those *x* ∈ [0,1] with a unique expansion *x* = ∑_*j*=0_^*∞*^ *x*_*j*_2^−*j*−1^ (almost all *x* ∈ [0,1] has such a unique expansion) by their associated binary sequence {*x*_*i*_}. For two such points *x*, *y* ∈ [0,1], define

(33) $$\begin{array}{c} x⊕y=\sum_{j=0}^{\infty }\left|x_{j}-y_{j}\right|2^{-j-1}. \end{array}$$

The operation ⊕ is defined for almost all *x*, *y* ∈ [0,1]. With this definition, we have

(34) $$\begin{array}{c} W_{n}\left(x⊕y\right)=W_{n}\left(x\right)W_{n}\left(y\right) \end{array}$$

for every pair *x*, *y* for which *x* ⊕ *y* is defined, (Golubov et al. [6], page 11).

## 3. Main Results

In this paper, three new theorems for the generalized Carleson operators on the periodic Walsh-type wavelet packets have been determined in the following form.

**Theorem 3**

Let $\left\{{\tilde{w}}_{n}\right\}$ be a periodic Walsh-type wavelet packet basis. Then for every *q*-block *β*, 1 < *q* ≤ *∞*,

(35) $$\begin{array}{c} \left|\left\{{𝔾}_{c}^{d}\beta >\alpha \right\}\right|\leq \frac{C_{q}}{\alpha },\;\alpha >0, \end{array}$$

where *𝔾*_*c*_^*d*^ is the generalized dyadic Carleson operator defined by (28) and *C*_*q*_ is a positive finite constant.

**Theorem 4**

Let $\left\{{\tilde{w}}_{n}\right\}$ be a periodic Walsh-type wavelet packet basis. Then for every *q*-block *β*, 1 < *q* ≤ *∞*,

(36) $$\begin{array}{c} \left|\left\{G_{c}\beta >\alpha \right\}\right|\leq \frac{C_{q}}{\alpha },\;\alpha >0, \end{array}$$

where *G*_*c*_ is the generalized weak Carleson operator defined by (26) and *C*_*q*_ is a positive finite constant.

**Theorem 5**

If a function *f* belongs to *𝔹*_*q*_-class, 1 < *q* ≤ *∞*, then

(37) $$\begin{array}{c} \left|\left\{G_{c}f>\alpha \right\}\right|=O\left({||f||}_{{𝔹}_{q}}\right),\;for\;\;\alpha >0, \end{array}$$

where *G*_*c*_ is the generalized weak Carleson operator.

## 4. Lemmas

For the proof of our theorems, the following lemmas are required.

**Lemma 6 (Nielsen [7])**

Let *f*_1_ ∈ *L* ^2^(ℝ), and define {*f*_*n*_}_*n*≥2_ recursively by

(38) $$\begin{array}{c} f_{2n}\left(x\right)=f_{n}\left(2x\right)+f_{n}\left(2x-1\right),\\ f_{2n+1}\left(x\right)=f_{n}\left(2x\right)-f_{n}\left(2x-1\right). \end{array}$$

Then

(39) $$\begin{array}{c} f_{n}\left(x\right)=\sum_{s=0}^{2^{J}-1}W_{n-2^{J}}\left(s2^{-J}\right)f_{1}\left(2^{J}x-s\right), \end{array}$$

where *n*, *J* ∈ *ℕ*, 2^*J*^ ≤ *n* < 2^*J*+1^.

**Lemma 7 (Zygmund [8], page 3)**

Consider

(40) $$\begin{array}{c} \sum_{\nu =1}^{n}u_{\nu }v_{\nu }=\sum_{\nu =1}^{n-1}\left(v_{\nu }-v_{\nu +1}\right)U_{\nu }+U_{n}v_{n}, \end{array}$$

where *U*_*k*_ = *u*_1_ + *u*_2_ + ⋯+*u*_*k*_ for *k* = 1,2,…, *n*; it is also called Abel's transformation.

**Lemma 8**

Let {*W*_*n*_}_*n*=0_^*∞*^ be the Walsh system. Then

(41) |∑n=2Km(1−nm−2k+1)Wn−2K([2Kx]2−K)    ×Wn−2K([2Ky]2−K)|  ≤Cx⊕y,

where *C* is a finite positive constant, *K* ≥ 1, 2^*K*^ ≤ *n* < 2^*K*+1,^ and for all pairs *x*, *y* ∈ [0,1) for which *x* ⊕ *y* is defined.

**Proof**

The Dirichlet kernel, *D*_*n*_(*x*) = ∑_*k*=0_^*n*−1^ *W*_*k*_(*x*), for the Walsh system satisfies

(42) $$\begin{array}{c} \left|D_{n}\left(x⊕y\right)\right|\leq \frac{1}{x⊕y} \end{array}$$

(see Golubov et al. [6], page 21).

Hence,

(43) |∑n=2Km(1−nm−2k+1)Wn−2K([2Kx]2−K)  ×Wn−2K([2Ky]2−K)| =|∑n=2Km(1−nm−2k+1)Wn−2K    ×([2Kx]2−K⊕[2Ky]2−K)| =|∑n=0m−2K(1−nm−2k+1)Wn    ×([2Kx]2−K⊕[2Ky]2−K)| =|∑n=0m−2K−1{(1−nm−2K+1)−(1−n+1m−2K+1)}     ×∑r=0nWr([2Kx]2−K⊕[2Ky]2−K)   +(1−m−2Km−2K+1)   ×∑n=0m−2KWn([2Kx]2−K⊕[2Ky]2−K)|,  by  Lemma  7, =|∑n=0m−2K−11m−2K+1   ×∑r=0nWr([2Kx]2−K⊕[2Ky]2−K)+1m−2K+1   ×∑n=0m−2KWn([2Kx]2−K⊕[2Ky]2−K)| ≤|∑n=0m−2K−1Wn([2Kx]2−K⊕[2Ky]2−K)+1m−2K+1   ×∑n=0m−2KWn([2Kx]2−K⊕[2Ky]2−K)| =|∑n=2Km−1Wn−2K([2Kx]2−K⊕[2Ky]2−K)+1m−2K+1   ×∑n=2KmWn−2K([2Kx]2−K⊕[2Ky]2−K)| ≤|∑n=2Km−1Wn−2K([2Kx]2−K⊕[2Ky]2−K)|+1m−2K+1   ×|∑n=2KmWn−2K([2Kx]2−K⊕[2Ky]2−K)| =|W2K([2Kx]2−K⊕[2Ky]2−K)Dm−2K   ×([2Kx]2−K⊕[2Ky]2−K)|+1m−2K+1  ×|W2K([2Kx]2−K⊕[2Ky]2−K)Dm−2K+1    ×([2Kx]2−K⊕[2Ky]2−K)| =|Dm−2K(x⊕y)|  +1m−2K+1|Dm−2K+1(x⊕y)| ≤1(x⊕y)+1m−2k+11(x⊕y), x⊕y≠0 =(1+1m−2k+1)1(x⊕y) ≤C(x⊕y),

where (32), (34), and the fact that *D*_*ν*+1−2^*K*^_ is a constant on dyadic intervals of the form [*l*2^−*K*^, (*l* + 1)2^−*K*^) are used. This completes the proof of Lemma 8.

**Lemma 9**

If

(44) KJ,m(σ)(x,y)=∑n=2Jm(1−nm−2J+1)wn(x)wn(y),            for    2J≤m<2J+1,

then

(45) $$\begin{array}{c} \left|K_{J,m}^{\left(\sigma \right)}\left(x,y\right)\right|\leq \sum_{l=-2N}^{2N}\frac{C}{\left|x-y+2^{K-J}l\right|}, \end{array}$$

where *C* is an arbitrary constant.

**Proof**

The kernel can be expanded as

(46) KJ,m(σ)(x,y) =∑n=2Jm(1−nm−2J+1)wn(x)wn(y) =∑n=2Jm(1−nm−2J+1)   ×(∑l=02J−K−1Wn−2J−K(l2−(J−K))w2K(2J−Kx−l)      ×∑k=02J−K−1Wn−2J−K(k2−(J−K))         ×w2K(2J−Ky−k)),  by  Lemma  6, =∑l=02J−K−1 ‍∑k=02J−K−1{∑n=2Jm(1−nm−2J+1)           ×(Wn−2J−K(l2−(J−K))              ×Wn−2J−K(k2−(J−K)))           ×w2K(2J−Kx−l)          ×w2K(2J−Ky−k)}.

Therefore, using Lemma 8,

(47) |KJ,m(σ)(x,y)|≤ ∑l=−NN′∑k=−NN′|∑n=2Jm(1−nm−2J+1)        ×Wn−2J−K([2J−K(x+2K−Jl)]2−(J−K))        ×Wn−2J−K([2J−K(y+2K−Jk)]2−(J−K))|      ×||w2K||∞2≤∑l=−NN′∑k=−NN′C(x+2K−Jl)⊕(y+2K−Jk),

where ∑′ indicates that only the terms for which *x* + 2^*K*−*J*^ *l* ∈ [0,1) and *y* + 2^*K*−*J*^ *k* ∈ [0,1), respectively, should be included in the sum. This implies the estimate

(48) $$\begin{array}{c} \left|K_{J,m}^{\left(\sigma \right)}\left(x,y\right)\right|\leq \sum_{l=-N}^{N}\;\sum_{k=-N}^{N}\frac{\tilde{C}}{\left|x-y+2^{K-J}\left(l-k\right)\right|}, \end{array}$$

since *a* ⊕ *b* ≥ 2^−log⁡_2_[|*a*−*b*|]^ ≥ |*a* − *b* | /2. This completes the proof of Lemma 9.

## 5. Proof of Theorem 3

The dyadic arithmetic mean of partial sums for the expansion of a measurable (integrable) function *f* in the periodic Walsh-type wavelet packets,

(49) (σ2Nf)(x)=12N∑n=02N−1(Snf)(x)=12N∑n=02N−1(∑k=0n〈f,w~k〉w~k(x)), by  (22),=∑n=02N−1(1−n2N)〈f,w~n〉w~n(x),

holds everywhere with the arithmetic mean of the projection onto the (periodized) scaling space ${\tilde{V}}_{N}$ associated with the underlying multiresolution analysis (Hess-Nielsen and Wickerhauser [9]). Therefore, it suffices to consider the arithmetic mean of the projection operators $P_{{\tilde{V}}_{N}}$ on to the space ${\tilde{V}}_{N}$.

Suppose that the *q*-block *β* is associated with the dyadic interval *I* ⊂ [0,1). If 1 < *α*|*I*|, then |*I*|^1−*q*^/*α* ^*q*^ ≤ 1/*α*, and using the fact that the operator $f\to \sup_{N}\sum_{n=0}^{N}\left(1-n/\left(N+1\right)\right)P_{{\tilde{V}}_{n}}f(x)$ (and thus *f* → *𝔾*_*c*_^*d*^ *f*(*x*)) is of strong type (*q*, *q*). We have

(50) $$\begin{array}{c} \left|\left\{{𝔾}_{c}^{d}f\left(x\right)>\alpha \right\}\right|\leq C_{q}{\left(\frac{{||\beta ||}_{q}}{\alpha }\right)}^{q}\leq C_{q}\frac{{\left|I\right|}^{1-q}}{\alpha^{q}}\leq \frac{C_{q}}{\alpha }. \end{array}$$

Now suppose that 1 ≥ *α*|*I*| with *I* = [*a*, *b*). Put $\tilde{I}=\left[(3a-b)/2,(3b-a)/2\right]\cap [0,1)$, and define $\overset{-}{I}=[0,1)-\tilde{I}$. We have

(51) |{𝔾cdf(x)>α}|≤2|I|+|I~∩{𝔾cdf(x)>α}|≤2α+|I~∩{𝔾cdf(x)>α}|.

Fix $x\in \overset{-}{I}$, and let *K*_*N*_(*x*, *y*) denote the operator kernel associated with the projection operators $P_{{\tilde{V}}_{N}}$. Then there exists a finite constant *C* (independent of *N*) such that

(52) $$\begin{array}{c} \left|K_{N}\left(x,y\right)\right|\leq \frac{C}{\left|x-y\right|} \end{array}$$

(see Terence [10]). 

Using the estimate (52) on the kernel *K*_*N*_, we obtain

(53) |(σ2Nβ)(x)| =|∑n=02N−1(1−n2N)〈β,w~n〉w~n(x)| =|∑n=02N−2{(1−n2N)−(1−n+12N)}   ×∑r=0n〈β,w~r〉w~r(x)   +(1−2N−12N)∑n=02N−1〈β,w~n〉w~n(x)|,   by  Lemma  7, =|∑n=02N−212N∑r=0n〈β,w~r〉w~r(x)   +12N∑r=02N−1〈β,w~n〉w~n(x)| =|∑n=02N−212N(Snβ)(x)+12N(S2Nβ)(x)| ≤∑n=02N−212N|(Snβ)(x)|+12N|(S2Nβ)(x)| ≤∑n=02N−212N|∫IKn(x,y)β(y)dy|  +12N|∫IK2N(x,y)β(y)dy| ≤∑n=02N−212N(C|x−a|+C|x−b|)||β||1  +12N(C|x−a|+C|x−b|)||β||1 =(2N−12N+12N)(C|x−a|+C|x−b|)||β||1 =(C|x−a|+C|x−b|)||β||1.

Since ||*β*||_1_ ≤ 1 and $x\in \overset{-}{I}$ implies that |*x* − *a* | , |*x* − *b* | ≥|*I* | /2, therefore,

(54) |(σ2Nβ)(x)|≤{2C|I|+2C|I|}=4C|I|≤C~α.

Finally we obtain

(55) $$\begin{array}{c} \left|\left\{x\in \overset{-}{I}:\underset{N}{\sup}\left|\left(\sigma_{2^{N}}\beta \right)\left(x\right)\right|>\alpha \right\}\right|\leq \frac{\tilde{C}}{\alpha }, \end{array}$$

where $\tilde{C}$ is independent of *I* and *β* and hence Theorem 3 follows. 

## 6. Proof of Theorem 4

Fix *α* > 0 and a *q*-block *β* supported on the dyadic interval *I* ⊂ [0,1); two cases are considered.

**Case I**

If 1 < *α* | *I*|, then |*I*|^1−*q*^/*α* ^*q*^ ≤ 1/*α*. Therefore, using Theorem 5.1. [7], page 275, we have

(56) |{Gcβ>α}|≤Cq(||β||qα)q≤Cq(|I|1/q−1)qαq=Cq|I|1−qαq≤Cqα.

**Case II**

Let 1 ≥ *α* | *I*| with *I* = [*a*, *b*). Let

(57) $$\begin{array}{c} \tilde{I}=\left(\cup_{j=-1}^{1}\left(j+\left[\frac{3a-b}{2},\frac{3b-a}{2}\right)\right)\right)\cap \left[0,1\right), \end{array}$$

and define $\overset{-}{I}=\left[0,1\right)\setminus \tilde{I}$. Then

(58) |{Gcβ>α}|≤|I~| +|I−∩{Gcβ>α}|≤3|I|+|I−∩{Gcβ>α}|≤6α+|I−∩{Gcβ>α}|.

Notice that

(59) |I−∩{Gcβ>α}|≤|I~∩{Gcdβ>α2}|+|I−∩{limsup⁡J MJβ>α2}|,

with

(60) $$\begin{array}{c} M_{J}\beta \left(x\right)=\underset{2^{J}\leq m<2^{J+1}-1}{\max}\;M_{J}^{m}\beta \left(x\right),\\ M_{J}^{m}\beta \left(x\right)=\left|\sum_{n=2^{J}}^{m}\left(1-\frac{n}{m-2^{J}+1}\right)\langle \beta ,{\tilde{w}}_{n}\rangle {\tilde{w}}_{n}\left(x\right)\right|. \end{array}$$

For *x* ∈ [0,1), we have

(61) limsup⁡J,m MJmβ(x)=limsup⁡J,m|∑n=2Jm(1−nm−2J+1)〈β,w~n〉w~n(x)|=limsup⁡J,m|∑l1=−NN ∑l2=−NN ∑n=2Jm(1−nm−2J+1)              ×〈β,wn(·−l1)〉w(x−l2)|≤∑l1=−NN ∑l2=−NNlimsup⁡J,m|∑n=2Jm(1−nm−2J+1)            ×〈β,wn(·−l1)〉w(x−l2)|.

Hence, it suffices to estimate |*E*_*α*_^*l*_1_,*l*_2_^| with

(62) Eαl1,l2={x∈I−:limsup⁡J,m|∑n=2Jm(1−nm−2J+1)              ×〈β,wn(·−l1)〉w(x−l2)|>α}.

Fix *x* ∈ ℝ∖*I*; then

(63) |∫−∞∞KJ,mσ(x−l1,y−l2)β(y)dy|  ≤C~∑l=−2N2N∫−∞∞β(y)dy|x−y+l2−l1+2K−Jl|,

which implies that whenever *x* ∈ *E*_*α*_^*l*_1_,*l*_2_^, there is an increasing sequence *J*_*k*_ → *∞* for which

(64) (1|x−a+l2−l1+2K−Jkl| +1|x−b+l2−l1+2K−Jkl|)>Cα,

for some fixed *C* > 0 and for *k* = 1,2,…. Since *J*_*k*_ → *∞*, therefore

(65) $$\begin{array}{c} \left(\frac{1}{\left|x-a+l_{2}-l_{1}\right|}+\frac{1}{\left|x-b+l_{2}-l_{1}\right|}\right)>C\alpha . \end{array}$$

Using that $\overset{-}{I}=[0,1)\setminus \tilde{I}$ and the same technique as in the proof of Lemma 9, we complete the proof to conclude that |*E*_*α*_^*l*_1_,*l*_2_^| ≤ 1/*α* and consequently

(66) $$\begin{array}{c} \left|\overset{-}{I}\cap \left\{\underset{J}{\mathrm{limsup}}M_{J}\beta >\frac{\alpha }{2}\right\}\right|\leq \frac{\tilde{C}}{\alpha }, \end{array}$$

which completes the proof of Theorem 4.

## 7. Proof of Theorem 5

Let *f* = ∑_*k*=1_^*∞*^ *c*_*k*_ *β*_*k*_ be a function of *𝔹*_*q*_. Then

(67) σNf=∑n=0N(1−nN+1)〈f,w~n〉w~n=∑n=0N(1−nN+1)〈∑k=1∞ckβk,w~n〉w~n=∑n=0N(1−nN+1)∑k=1∞ck〈βk,w~n〉w~n=∑k=1∞ck(∑n=0N(1−nN+1)〈βk,w~n〉w~n)=∑k=1∞ck(σNβk),

due to the *L* ^1^ convergence of the average sum defining *f*. Since

(68) ∑n=0N(1−nN+1)〈f,w~n〉w~n =∑k=1∞ck∑n=0N(1−nN+1)〈βk,w~n〉w~n,limsup⁡N|∑n=0N(1−nN+1)〈f,w~n〉w~n| ≤∑k=1∞|ck|limsup⁡N|∑n=0N(1−nN+1)〈βk,w~n〉w~n|Gcf≤∑k=1∞|ck|‍Gcβk

therefore

(69) |{Gcf>α}|≤|{∑k=1∞|ck|Gcβk>α}|≤Cqα∑k=1∞|ck|, by  Theorem  4,≤Cqα||f||𝔹q, by  (12),=O(||f||𝔹q).

This completes the proof of Theorem 5. 

## 8. Applications

Following corollary can be deduced from our theorems.

**Corollary 10**

Let $\left\{{\tilde{w}}_{n}\right\}$ be a periodic Walsh-type wavelet packet basis. Then the Fourier expansion of any function *f* ∈ *𝔹*_*q*_, 1 < *q* < *∞*, in $\left\{{\tilde{w}}_{n}\right\}$ is summable by arithmetic means pointwise a.e.

**Proof**

Let us write $\left(S_{N}f)(x)=\sum_{n=0}^{N}\langle f,{\tilde{w}}_{n}\rangle {\tilde{w}}_{n}(x\right)$ and

(70) (σNf)(x)=1N+1∑n=0N(Snf)(x)=∑n=0N(1−nN+1)〈f,w~n〉w~n(x).

With *f* = ∑_*k*=1_^*∞*^ *c*_*k*_ *β*_*k*_ ∈ *𝔹*_*q*_, let *g*_*K*_ = ∑_*k*=1_^*K*^ *c*_*k*_ *β*_*k*_, and observe that ||*f*−*g*_*K*_||_*𝔹*_*q*__ → 0. For each *x* ∈ [0,1), write

(71) f−σNf=(f−gK)+(gK−σNgK)+(σNgK−σNf).

Thus

(72) |{x:limsup⁡N→∞|f(x)−(σNf)(x)|>α}|≤|{x:limsup⁡N→∞|f(x)−gK(x)|>α3}| +|{x:limsup⁡N→∞|gK(x)−(σNgK)(x)|>α3}| +|{x:limsup⁡n→∞|(σNgK)(x)−(σNf)(x)|>α3}|≤|{x:limsup⁡N→∞|f(x)−gK(x)|>α3}| +|{x:limsup⁡N→∞|gK(x)             −∑n=0N(1−nN+1)               ×〈gK,w~n〉w~n(x)|>α3}| +|{x:limsup⁡N→∞|∑n=0N(1−nN+1)             ×〈gK−f,w~n〉w~n(x)|>α3}|≤|{x:limsup⁡N→∞|f(x)−gK(x)|>α3}| +|{x:limsup⁡N→∞|gK(x)−∑n=0N〈gK,w~n〉w~n(x)|>α3}| +|{x:limsup⁡N→∞|∑n=0N〈gK−f,w~n〉w~n(x)|>α3}|≤|{x:limsup⁡N→∞|f(x)−gK(x)|>α3}| +|{x:limsup⁡N→∞|gK(x)−(SNgK)(x)|>α3}| +|{x:limsup⁡N→∞|(SNgK)(x)−(SNf)(x)|>α3}|≤3α||f−gK||𝔹q+0+3αCq||f−gK||𝔹q, by  Theorem  5.

From this it follows that

(73) $$\begin{array}{c} \left|\left\{x:\underset{n\to \infty }{\mathrm{limsup}}\left|f\left(x\right)-\left(\sigma_{N}f\right)\left(x\right)\right|>\alpha \right\}\right|=0. \end{array}$$

This completes the proof of the corollary.
